# Estimating measures of latent variables from m-alternative forced choice responses

**DOI:** 10.1371/journal.pone.0225581

**Published:** 2019-11-22

**Authors:** Chris Bradley, Robert W. Massof

**Affiliations:** Department of Ophthalmology, Johns Hopkins University School of Medicine, Baltimore, Maryland, United States of America; Universitat de Valencia, SPAIN

## Abstract

Signal Detection Theory is the standard method used in psychophysics to estimate person ability in *m*-alternative forced choice tasks where stimuli are typically generated with known physical properties (e.g., size, frequency, contrast, etc …) and lie at known locations on a physical measurement axis. In contrast, variants of Item Response Theory are preferred in fields such as medical research and educational testing where the axis locations of items on questionnaires or multiple choice tests are not defined by any observable physical property and are instead defined by a latent (or unobservable) variable. We provide an extension of Signal Detection Theory to latent variables that employs the same strategy used in Item Response Theory and demonstrate the practical utility of our method by applying it to a set of clinically relevant face perception tasks with visually impaired individuals as subjects. A key advantage of our approach is that Signal Detection Theory explicitly models the *m*-alternative forced choice task while Item Response Theory does not. We show that Item Response Theory is inconsistent with key assumptions of the *m*-alternative forced choice task and is not a valid model for this paradigm. However, the simplest Item Response Theory model–the dichotomous Rasch model–is found to be a special case of SDT and provides a good approximation as long as the number of response alternatives *m* is small and remains fixed for all items.

## Introduction

In typical psychophysics experiments, stimuli are generated with a known physical property (e.g., size, frequency, contrast, etc …) that defines the locations of these stimuli on a physical measurement axis. Psychometric functions fit to subject responses in tasks involving these stimuli allow researchers to estimate person abilities in corresponding physical stimulus units (e.g., "thresholds" or their inverse: "sensitivities"). However, there are many tasks where it is necessary to use stimuli whose locations on the measurement axis are defined by a latent (or unobservable) variable–there is no known physical property of the stimuli that defines their locations on the measurement axis. For example, it is of clinical importance to measure the ability of visually impaired individuals to identify common objects or recognize other people, and it is not a priori clear where on the measurement axis an image of an object or person should be placed. The aim of this paper is to extend psychophysics to cases where the locations of "items" (stimuli or tasks) on the measurement axis must first be estimated from subject response data before estimating person ability. Our proposed solution applies specifically to the case where the experimental paradigm is *m*-alternative forced choice (*m*-AFC)–on each trial there are *m*≥2 possible response choices with precisely one choice defined as "correct" and all others defined as "incorrect" by the experimenter.

In fields ranging from medical research to educational testing, a popular solution to the problem of estimating "item measures" (locations of items on the measurement axis) from subject responses is to use some variant of Item Response Theory (IRT) [1–4]. The main problem with using IRT to extend psychophysics to cases where item locations are defined by a latent variable is that IRT models specify the probability of observing an outcome without representing the underlying task, which makes it difficult to determine which tasks each IRT model applies to. For example, in medical research the most common application of IRT is the analysis of responses to health status questionnaires where each person rates items on an ordinal rating scale, while in educational testing IRT is often applied towards analyzing responses to multiple choice tests where every item is a *m*-AFC task. These two types of tasks are fundamentally different because a person's rating of an item is not scored correct or incorrect by the experimenter or test giver, while the observed score in a *m*-AFC task is the result of comparing a person's response to an item to a defined truth state. Yet the same IRT model is applied to both tasks.

It is important to prove, or at least to derive from precise assumptions, that a given model applies to a given task. For example, a specific variant of IRT has been shown to be the logically implied model from generally agreed upon assumptions about how a person rates an item on a given trial [5], which include the mathematical definition of a rating scale–a real line partitioned by ordered thresholds (points on the real line) into ordered intervals called rating categories–as well as commonly held assumptions about trial to trial variability in person ability, item difficulty and threshold locations. Yet no comparable derivation of an IRT model exists from a model of *m*-AFC tasks, and it is unclear whether IRT models apply to tasks where a person's response is compared to an underlying truth state to generate a score of "correct" or "incorrect". In psychophysics, there is a long history of using Signal Detection Theory (SDT) to model *m*-AFC tasks [6], and we will show that SDT can be extended to cases where item locations on the measurement axis are defined by a latent variable–we will henceforth refer to this as "extending SDT to latent variables"–using the same strategy employed in IRT models. Our extension of SDT to latent variables is specifically tailored to the *m*-AFC paradigm where the underlying truth state is known to the experimenter. Other extensions of SDT to latent variables apply to tasks where the underlying truth state is unknown and raters' responses are used to estimate the underlying truth state [7–9].

Our approach has several advantages over IRT. Many IRT models add an extra "guessing" parameter to deal with chance performance [3,10] while our method naturally incorporates chance performance without requiring ad hoc assumptions. Our method also estimates person ability on a scale that does not depend on which item a person is compared to while many IRT models have an "item discrimination" parameter that is specific to each item, acts as a scalar on the unit of measurement, and effectively allows each item to estimate person ability on its own scale. One consequence of this item discrimination parameter is that IRT models incorporating it do not satisfy a Guttman scale [4], which is a fundamental property of measurement that says that all items must agree in their ordering of the persons and all persons must agree in their ordering of the items. The simplest IRT model–the dichotomous Rasch model–does satisfy a Guttman scale and we will show that the dichotomous Rasch model is a mathematically special case of SDT. The practical utility of our method will be demonstrated by applying it to a set of clinically relevant face perception tasks with visually impaired individuals as subjects.

## Methods

### Extending SDT to latent variables

SDT models the *m*-AFC task by postulating the existence of separate internal responses (e.g., an internal cognitive decision variable in response to the stimuli) for each of the *m* possible response choices presented by the item–precisely one of these *m* possible response choices is defined to be "correct" by the experimenter. While each of these *m* internal responses is in principle a separate internal decision variable, SDT concerns itself only with the magnitudes of these internal responses and places them on the same axis, which we will call the *x*-axis. Whichever response choice generates the largest (not necessarily correct) internal response is assumed to be the response chosen by the person on that trial. Probability correct equals the probability that the defined correct choice generates an internal response greater than all *m*−1 internal responses to the incorrect choices. The general solution to this problem is

$$p\left(C\right)=\int_{-\infty }^{\infty }f_{C}\left(x\right)\prod_{k=1}^{m-1}F_{I,k}\left(x\right)dx$$ (1)

where *p*(*C*) represents probability correct, *f*_*C*_(*x*) represents the probability density function of the magnitude of the internal response to the correct choice, and *F*_*I*,*k*_(*x*) represents the cumulative distribution function of *f*_*I*,*k*_(*x*) which is the analog to *f*_*C*_(*x*) for the *k*th incorrect choice, for *k*∈{1,…,*m*−1}. The main problem with Eq 1 is that neither *f*_*C*_(*x*) nor *f*_*I*,*k*_(*x*), for any *k*, are in general known and SDT makes the simplifying assumption that *f*_*I*,*k*_(*x*)~*N*(*μ*_1_,*σ*) for all *k* and *f*_*C*_(*x*)~*N*(*μ*_2_,*σ*), allowing us to use $d\prime =\frac{\mu_{2}-\mu_{1}}{\sigma }$, or "d prime", as a practical unit of measurement between *f*_*C*_(*x*) and *f*_*I*,*k*_(*x*) for any *k*. Because *d*′ is in standard deviation units and the axis origin is arbitrary, we can set *μ*_1_ = 0 and *σ* = 1 without loss of generality and turn Eq 1 into the more conventional

$$p\left(C\right)=\int_{-\infty }^{\infty }\varphi \left(x-d^{\prime }\right){\left[\Phi \left(x\right)\right]}^{m-1}dx$$ (2)

where *φ*(*x*) is the standard normal distribution and Φ(*x*) is its cumulative distribution function [6,11].

To extend SDT to latent variables we adopt a strategy similar to the one employed by IRT models. To illustrate, consider the dichotomous Rasch model (a 1-parameter IRT model), which is the simplest IRT model and permits only two possible responses that we will represent as 0 and 1 [12]. If we represent the response of person *i* to item *h* as *R*_*ih*_∈{0,1}, then the dichotomous Rasch model assumes that the probability of observing *R*_*ih*_ = 1 is related to person measure *θ*_*i*_ and item measure *b*_*h*_ (estimates of person ability and item difficulty, respectively, using conventional IRT notation) through the logistic function: $p(R_{ih}|\theta_{i},b_{h})=e^{(\theta_{i}-b_{h})R_{ih}}/\left(1+e^{\left(\theta_{i}-b_{h}\right)}\right)$. To estimate all item and person measures at desired levels of precision, responses from a sufficiently large number of persons to the same set of items are obtained through a maximum likelihood estimation (MLE). Unlike typical psychophysics experiments, IRT models are generally applied to the responses of a large number of persons (hundreds or even thousands of subjects is common) with each person responding at most once to each item.

If computational constraints were not an issue, we could extend SDT to latent variables with a MLE using the following likelihood function based on Eq 2:

$$L_{m}\left(\theta_{i}-b_{h}\right)=\int_{-\infty }^{\infty }\varphi \left(x-\left(\theta_{i}-b_{h}\right)\right){\left[\Phi \left(x\right)\right]}^{m-1}dx$$ (3)

where we set *d*′ = *θ*_*i*_−*b*_*h*_ and make the likelihood *L*_*m*_(*θ*_*i*_−*b*_*h*_) of the response by person *i* to item *h* dependent on the number of response alternatives *m*. A MLE using Eq 3 would find the set of person and item measures that maxmizes the likelihood ∏_*i*,*h*_*L*_*m*_(*θ*_*i*_−*b*_*h*_) of observing the set of responses from all persons *i* to all items *h*.

Conceptually, IRT models estimate item measures relative to the sample of persons, suggesting that a more computationally tractable estimation method (than a MLE) begin with estimating all item measures by treating all responses as repeated measures from a single "average" person. Specifically, let *p*_*h*_(*C*) represent probability correct for item *h* relative to the sample of persons and define the person measure of the "average" person to be *θ* = 0. Setting *θ* = 0, we can estimate a ${\hat{b}}_{h}$ item measure for item *h* independently of all other items by solving

$$p_{h}\left(C\right)=\int_{-\infty }^{\infty }\varphi \left(x+b_{h}\right){\left[\Phi \left(x\right)\right]}^{m-1}dx$$ (4)

Confidence intervals on item measures can be calculated by mapping binomial confidence intervals (the endpoints of which are in "probability correct" units) into *d*′ units through Eq 4 –we used the Wilson method for calculating binomial confidence intervals [13].

Once all item measures are estimated, each person measure can be independently estimated through a MLE, which is computationally tractable because there is only one parameter being estimated at any time. Let *A*_*i*,*m*_ represent the set of item measures corresponding to every correct response person *i* made to a *m*-AFC item, and let *B*_*i*,*m*_ represent the set of item measures corresponding to every incorrect response made by person *i* to a *m*-AFC item. Then estimated person measure ${\hat{\theta }}_{i}$ is the solution to

$${\hat{\theta }}_{i}=\underset{\theta }{\arg\;\max}\left(\sum_{m}\left[\sum_{b\in A_{i,m}}\log\left(L_{m}\left(\theta -b\right)\right)+\sum_{b\in B_{i,m}}\log\left(1-L_{m}\left(\theta -b\right)\right)\right]\right)$$ (5)

Because a MLE was used, person measure standard errors are the reciprocal of the square root of the Hessian.

An Expectation Maximization (EM) approach that estimates a "local" MLE is also possible by iteratively estimating item and person measures after an initial set of item and person measures is estimated using Eqs 4 and 5. In this iterative process, item measures are estimated given the most recently estimated person measures and person measures are estimated given the most recently estimated item measures, until the difference between estimated parameters from successive iterations falls below a desired threshold. Eq 5 can be used to estimate person measures in this iterative process. However, a new equation is needed for estimating item measures:

$${\hat{b}}_{h}=\underset{b}{\arg\;\max}\left(\sum_{\theta \in A_{h}}\log\left(L_{m}\left(\theta -b\right)\right)+\sum_{\theta \in B_{h}}\log\left(1-L_{m}\left(\theta -b\right)\right)\right)$$ (6)

where ${\hat{b}}_{h}$ is the estimated item measure for a *m*-AFC item, *A*_*h*_ is the set of person measures associated with every correct response to item *h*, and *B*_*h*_ is the set of person measures associated with every incorrect response to item *h*. Code in R is provided for both approximation methods [14]. Estimated parameters from both methods were compared to each other using data from our facial expression discrimination experiment.

### Application to facial expression discrimination

To demonstrate how our extension of SDT to latent variables works in practice, we applied our method to three face perception tasks: 1) identifying a person's gender from three images of that person's face, 2) determining which of those three images shows a facial expression different from the other two (an "odd one out" task), and 3) identifying the emotional expression of the image the subject chose as the "odd one out". All subjects in our experiment were visually impaired individuals, and all three face perception tasks were of clinical relevance as many visually impaired individuals consider these tasks to be both important and difficult. We also tested subjects in two different magnification conditions, "with magnification" and "without magnification", which allowed us to develop a clinical outcome measure for low vision enhancement.

**Fig 1:**
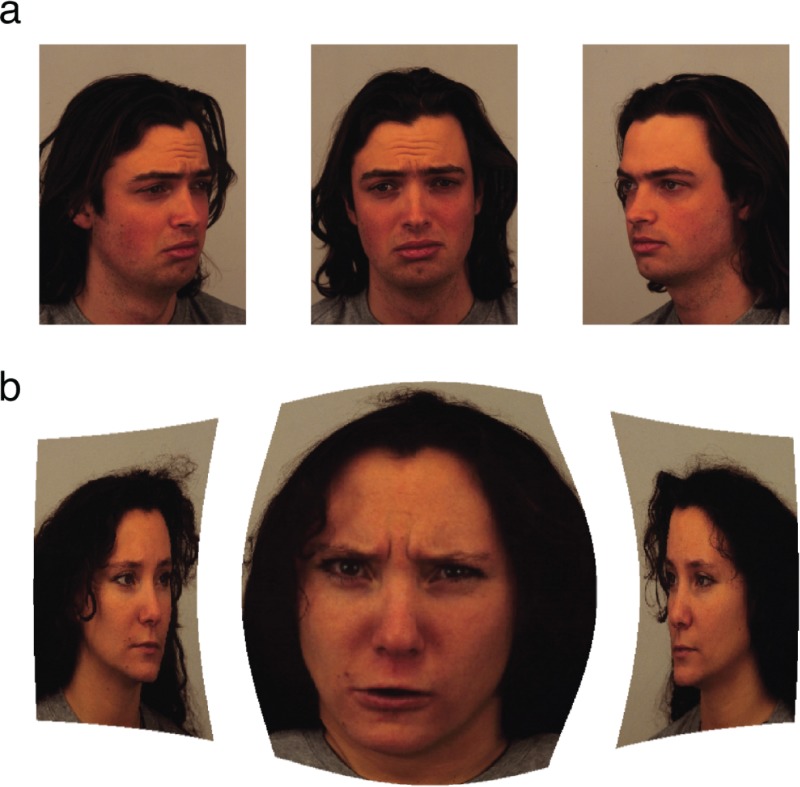
Example of stimuli presented in the head mounted display. A triplet of images (left, center, right) from the Karolinska Directed Emotional Faces database was presented on each trial in virtual reality at a fixed virtual distance using the Oculus DK2. Subjects could use head movements to center their gaze on any of the three images. Fig 1A shows an example of images presented in the "without magnification" condition and Fig 1B shows an example of images presented in the "with magnification" condition where a virtual bioptic telescope whose size, shape and region of magnification was customized to the patient (shown with gaze centered on the middle image).

A total of 50 visually impaired subjects (27 female, 23 male) were recruited from the Johns Hopkins low vision clinic with the inclusion criteria being that the subjects' best corrected visual acuity in the better seeing eye was between 20/60 and 20/800. Most subjects had either age-related macular degeneration or Stargardt’s disease (a.k.a. juvenile macular degeneration), the median best corrected visual acuity in the better seeing eye was 20/200 and the median age was 51 (16–91). On each trial, subjects were presented with three different views of the same person's face (Fig 1A) in virtual reality and at a fixed virtual distance using an Oculus DK2 head mounted display (HMD). Subjects could use head movements to center their gaze on any of the three images. Two of the images showed the same emotional expression while the third showed a different emotional expression (the "odd one out" image). Only three emotional expressions were presented in our experiment, "angry", "sad" and "neutral", with all images taken from and labeled in the Karolinska Directed Emotional Faces (KDEF) database [15,16]. There were a total of 64 trials, and on each trial subjects answered three questions with no time limit: 1) what is the gender of the individual, 2) which of the three faces shows a different emotional expression from the other two, and 3) what is the emotional expression of the image you (the subject) chose as the odd one out. For purposes of analysis, each triplet of images was treated as a different item in each of the three face perception tasks. Thus, in total there were 64 triplets × 3 tasks = 192 items.

For each subject, precisely half the trials were in the "with magnification" condition where faces were viewed through a virtual bioptic telescope whose size, shape and level of magnification were customized to the subject (Fig 1B). The telescopic magnification remained centered in the image, thus magnifying whichever face the subject was centering [17]. To determine which trials were in the with magnification condition, we partitioned the 64 trials into 8 blocks of 8 trials each and randomly chose for each person either all odd numbered blocks or all even numbered blocks to be viewed with magnification. For purposes of analysis we assumed that there were 50 subjects × 2 magnification conditions = 100 "persons" with each subject being counted twice, once for each magnification condition. This allowed us to measure the effect of a simulated low vision enhancement intervention on each subject. Our experiment followed the tenets of the Declaration of Helsinki, informed consent was obtained from all subjects after explaining the nature and possible consequences of the study, and our research was approved by Johns Hopkins IRB. Original data from our experiment as well as R code for data analysis have been made available [14].

For many tasks, there are multiple possible SDT models–each represents a different set of assumptions about how humans represent the task and make decisions–and the SDT model chosen can change the calculation of *d*′ for the same data [18]. In general, it is not known which SDT model is accurate (if any) though for specific tasks (e.g., the same–different task) it has been shown that certain decision rules are qualitatively inconsistent with observed response probabilities [19]. For our three face perception tasks we made the plausible assumption that subjects have separate internal "detectors" for each of the *m* possible stimulus classifications (response choices) with each detector producing its own internal response. Thus, in the gender identification task we assumed that each subject had separate internal detectors for "male" and "female", and in the emotional expression identification task we assumed that each subject had separate internal detectors for "angry", "sad" and "neutral". The subject's response on any trial was determined by whichever internal response was largest.

Our assumptions are plausible but differ from those made in typical applications of SDT in psychophysics where only one "detector" is assumed to exist for a known signal in the presence of background noise. However, our tasks were not yes–no tasks where subjects are asked questions like "Is this person a male?" and the nature of the question suggests that only one "male" detector is needed. Our tasks required the subject to report the stimulus classification themselves and there is no a priori reason why a subject should only have a "male" detector and no "female" detector. If however the subject only used a single detector, then it is important to note that a criterion dependent SDT model must be applied, while our equations imply a criterion independent form of SDT. The question of whether a criterion dependent or criterion independent SDT model should be used is arguably less of an issue with our two other face perception tasks. It is possible to represent three different emotions on a single axis with two criteria partitioning the axis into the three emotions, but it is arguably more plausible to represent emotions in a multi-dimensional space. And with the odd one out task where we assumed the detectors were of the form "the image at location *x* is the odd one out", it may be impossible to model the task with a criterion dependent SDT model. We note that it is not necessary to specify how our hypothesized detectors work to calculate *d*′, but a plausible mechanism is through cross correlations, either between the stimulus and a template or in the case of the odd one out task between the two incorrect choices. Given these assumptions, our three face perception tasks reduce to *m*-AFC tasks, and we can estimate item and person measures either through Eqs 4 and 5 or through EM. We estimated both item and person measures for each task separately as well as for all three face perception tasks combined.

### Statistical analysis of data

To determine how good of an approximation Eqs 4 and 5 are to EM (a "local" MLE), we looked at *r*^2^ between item and person measures of both methods; mean absolute differences in *d*′ units were also calculated. For our face perception experiment, we looked at correlations between all pairs of estimated item measures to determine if the same items in different face perception tasks were measuring the same type of face perception ability. Correlations among estimated person measures were used to determine whether subjects who were good at one face perception task were also good at the others. The effect of magnification on face perception ability was determined through a paired t-test on estimated person measures.

## Results

We compared estimated item and person measures using Eqs 4 and 5 to parameters estimated through EM to see how well Eqs 4 and 5 approximate a local MLE. Parameters estimated from both methods on the combined data from all three face perception tasks were highly similar to each other with the mean absolute difference being 0.0278 *d*′ between the two sets of person measures and 0.1064 *d*′ between the two sets of item measures; *r*^2^>0.9996 for the persons and *r*^2^>0.9958 for the items. The larger discrepacy in both cases for the item measures was expected given that item measures were estimated before person measures. Because Eqs 4 and 5 provide a good approximation to a local MLE, all further analysis was done using this approximation method treating the sample of persons as a single "average" person.

**Fig 2:**
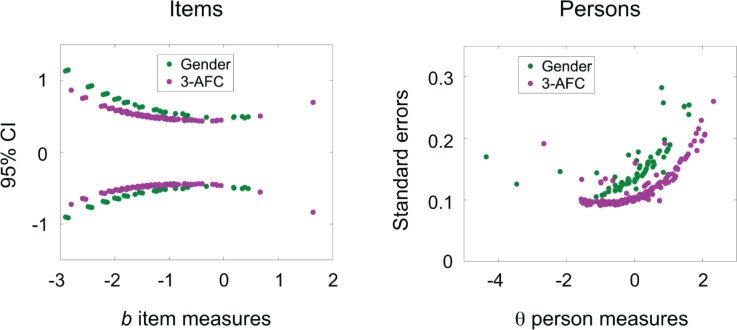
Confidence intervals for estimated item and person measures. Estimated item measures are plotted with their 95% CI (left), and estimated person measures are plotted with their standard errors (right), in *d*′ units. The data are color coded by 2-AFC (green) and 3-AFC (purple) and show that confidence intervals depend on the number of response alternatives.

Fig 2 plots the estimated $\hat{b}$ item measures of our face perception tasks together with their 95% CI (left) and the estimated $\hat{\theta }$ person measures for each subject together with their standard errors (right), all in *d*′ units. Item measures were directly estimated using Eq 4 with negative *b* representing easier items and *b* = 0 representing chance performance for the "average" person, for every *m*. Person measures were estimated from Eq 5 with *θ* representing the number of *d*′ units the sigmoid function defined by Eq 4 has to shift (on the *d*′ axis) to best fit that person's responses to the items. Since item measures were estimated from the average person, *θ* = 0 on the person measure plot represents the average person; more capable persons have more positive *θ* and less capable persons have more negative *θ*. Correlations among the item measures for the three face perception tasks were low: *r* = −0.08 between the gender identification task and the odd one out task, *r* = −0.21 between the gender identification task and emotional expression identification task, and *r* = 0.20 between the odd one out task and emotional expression identification task; this shows that the three face perception tasks measure different types of face perception ability. There were two items in the emotional expression identification task whose 95% CI were strictly above *b* = 0, meaning that for these two items in this task there was a statistically significant disagreement between the sample of persons and the labeling of emotional expressions in the KDEF database.

Fig 2 shows that both the 95% CI for the items and the standard errors for the persons depend on the number of response alternatives *m*. These results follow directly from Eqs 4 and 5 which both depend on *m*. The general arc-like patterns occur because precision decreases the farther away one moves from where most items are located (when estimating person measures) and where most persons are located (when estimating item measures). The 95% CI for the item measures exhibit "clumping" behavior because different numbers of persons (anywhere from 46 to 51) responded to different items, and binomial confidence intervals depend not only on the number of correct responses but also on the total number of responses. To give an example, the 4 item measures in the gender identification task (green dots) in the interval *b* = [−2.461,−2.421] have 4 different ratios of correct responses to total number of responses (trials): 47 correct out of 49, 46 out of 48, 45 out of 47, 44 out of 46. There is no smooth transition from this group to the neighboring group of 4 item measures in the interval *b* = [−2.213,−2.139] with ratios: 48 correct out of 51, 46 out of 49, 45 out of 48, and 43 out of 46.

**Fig 3:**
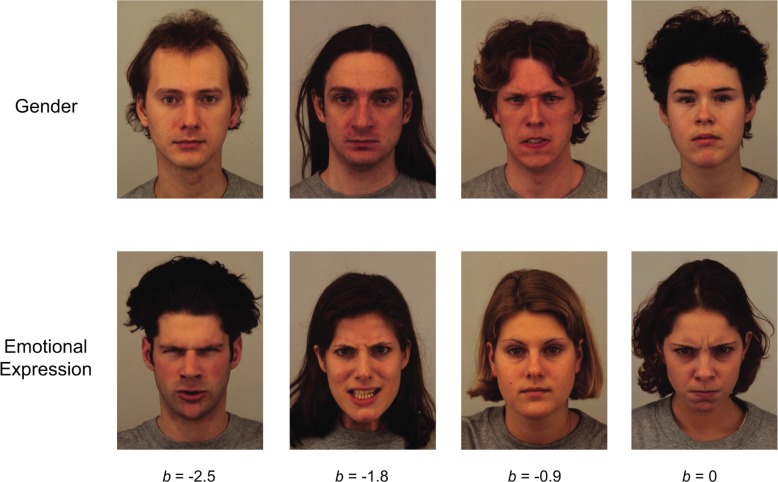
Examples of item difficulty for visually impaired individuals. Examples of item difficulty are shown for 4 items (taken from the Karolinska Directed Emotional Faces database) in the gender identification task (top row) and 4 items in the emotional expression identification task (bottom row). Only the center images of the triplet are shown in every case. Listed *b* item measures apply to both rows and are within 0.05 *d*′ units of the actual *b* except for the right-most column where the actual *b* was within 0.2 *d*′ units of listed. Negative *b* item measures represent easier items.

Fig 3 shows examples of just the center images (of the triplet) of easier to more difficult items for visually impaired individuals, with more negative *b* item measures representing easier items. The listed *b* item measures apply to both gender and emotional expression tasks and are within 0.05 *d*′ units of the actual *b* except for the right-most column where the deviation is at most 0.2 *d*′ units from listed. Several volunteers with normal vision who performed all three face perception tasks found the tasks fairly easy and rarely responded incorrectly. Thus, it is important to remember that these *b* item measures are specific to the sample of visually impaired individuals we tested.

**Fig 4:**
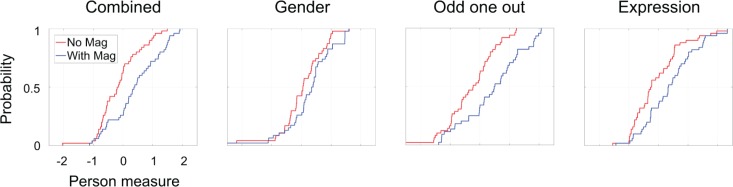
Cumulative distribution functions of person measures. Cumulative distribution functions of estimated person measures are shown for the "without magnification" condition (red) and for the "with magnification" condition (blue). Magnification improved performance in all tasks, with the average increase in person measure being Δ*θ* = 0.552 (*p*<10^−7^ using a paired t-test) across all tasks, Δ*θ* = 0.226 (*p*<0.035) for the gender identification task, Δ*θ* = 0.727 (*p*<10^−7^) for the odd one out task, and Δ*θ* = 0.452 (*p*<10^−4^) for the emotional expression identification task.

Fig 4 shows the cumulative distribution functions of person measures in the two magnification conditions, for all three face perception tasks combined (far left) and for each of the three face perception tasks separately. Magnification improved person measures by on average Δ*θ* = 0.552 (*p*<10^−7^ using a paired t-test) across all three face perception tasks, by Δ*θ* = 0.226 (*p*<0.035) for the gender identification task, by Δ*θ* = 0.727 (*p*<10^−7^) for the odd one out task, and by Δ*θ* = 0.452 (*p*<10^−4^) for the emotional expression identification task. We note that since *d*′ = *θ*−*b*, a change in *d*′ could be attributed to the person, to the item, or to both. Since our experiment tested the effect of magnification on face perception tasks for visually impaired individuals, our analysis assumed that the triplet of faces (the items) was invariant and that the magnification was applied to the visually impaired subject and thus changed the person measure. We also note that *d*′ comparisons across tasks depend on the accuracy of the SDT models used to estimate *d*′. If for example a criterion dependent SDT model is more accurate for the gender identification task while a criterion independent SDT model is more accurate for the odd one out task, then *d*′ for gender identification will depend on the distribution of criteria used across subjects while *d*′ for odd one out will not, and *d*′ will not be the same unit of measurement in the two tasks.

Person measures between the odd one out and emotional expression identification tasks were most highly correlated at *r* = 0.77, while the correlations between the other two pairs were lower: *r* = 0.31 between the gender identification and odd one out tasks, and *r* = 0.09 between the gender identification and the emotional expression identification tasks. These correlations were similar to the correlations observed when only looking at person measures in a given magnification condition: *r* = 0.78 (no magnification) and *r* = 0.77 (with magnification) between the odd one out and emotional expression identification tasks, *r* = 0.34 (no magnification) and *r* = 0.27 (with magnification) between the gender identification and odd one out tasks, and *r* = 0.07 (no magnification) and *r* = 0.20 (with magnification) between the gender identification and emotional expression identification tasks.

**Fig 5:**
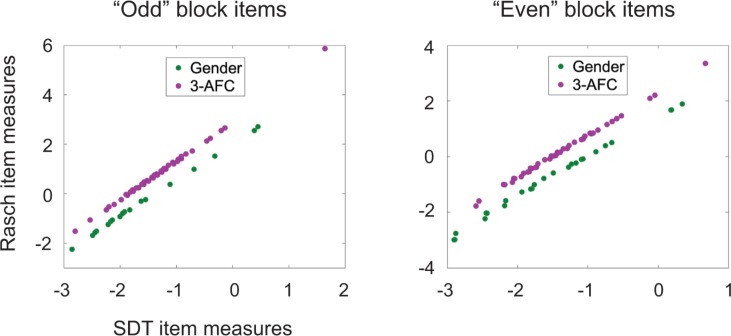
SDT vs. Rasch. Estimated item measures are shown for SDT in d prime units and for the dichotomous Rasch model in logits with "odd" block items (blocks 1, 3, 5 and 7) and "even" block items (blocks 2, 4, 6 and 8) plotted separately because different groups of persons responded to the two sets of items. For both groups the same relation holds: item measures are linearly related for any given *m*, but the intercepts of the best-fitting lines differ for different *m*. The slopes of the best-fitting lines for the 2-AFC task were nearly identical: 1.4782 for the "odd" items and 1.4737 for the "even" items; while the slopes differed for the 3-AFC tasks: 1.6064 for the "odd" and 1.5204 for the "even" items.

Fig 5 compares item measures estimated from our extension of SDT to those of the dichotomous Rasch model. Item measures were estimated from the combined data for all three face perception tasks. Data are plotted separately for "odd" block items (blocks 1, 3, 5 and 7) and "even" block items (blocks 2, 4, 6 and 8) because different persons responded to the two sets of items–this was a consequence of randomizing magnification conditions for each subject while treating the same subject as two different persons for the two magnification conditions. SDT item measures are plotted in *d*′ units with *b* = 0 representing chance performance for the average person for all *m*. Rasch item measures are plotted in logits on an axis whose origin (by convention) is at the mean item measure. For both "odd" and "even" block items, the same relation is observed: Rasch and SDT item measures are linearly related for any given value of *m*, but the intercepts of the best-fitting lines are different for different *m*. A closer look shows that the slopes of the best-fitting lines are essentially the same for the 2-AFC items (1.4782 for the "odd" block items and 1.4737 for the "even" block items) but differ more for the 3-AFC items (1.6064 for "odd" and 1.5204 for "even").

These results are explained by the dependency of Eq 4 (SDT) on *m*. When *m* = 2, Eq 4 produces a symmetric sigmoid function that is similar in shape to the logistic function of the dichotomous Rasch model, which leads to both the observed linear relation and the same predicted slope for the best-fitting lines. When *m*>2, Eq 4 produces an asymmetric sigmoid function, and the slopes of the best-fitting lines will depend on the set of estimated item measures. The intercepts differ primarily because the sigmoid functions defined in Eq 4 are shifted along the *d*′ axis so that chance performance (which is different for different *m*) is always mapped to *d*′ = 0, while the dichotomous Rasch model uses the same sigmoid function for all *m*.

## Discussion

We have presented an extension of SDT that estimates both item and person measures from *m*-AFC responses when item locations are defined by a latent variable. Unlike IRT models that specify the probability of observing an outcome without modeling the underlying task, our extension of SDT explicitly models both the cognitive processes underlying the *m*-AFC task as well as how the decision variable maps onto probability correct. There is currently no known way to directly test the accuracy of key assumptions of SDT such as its assumption that separate internal responses exist for each of the *m* choices, but there is support for such assumptions in current models of decision making such as the linear ballistic accumulator (LBA) [20]. The LBA predicts reaction time in a variety of *m*-AFC tasks, and it does so by assuming the existence of independent "accumulators" that repeatedly sample from what are essentially the hypothesized internal response distributions in SDT. Thus, LBA is a generalization of the model presented here, and it applies to stimuli defined by a latent variable. Our innovation with respect to models like LBA is that our extension of SDT applies to cases where both item and person measures are defined by a latent variable, while LBA measures person ability on the physical measurement axis of reaction time.

One advantage of our extension of SDT when compared to IRT is that it naturally incorporates chance performance. Many IRT models incorporate chance performance by adding an extra "guessing" parameter which provides a lower asymptote on performance [3,10]. Adding such a parameter may be justifiable when it is a priori clear that a person cannot asymptotically perform below chance (e.g., this occurs in typical psychophysics experiments where stimuli with known physical properties are presented and it is physically impossible for any observer to distinguish between the "correct" choice and all "incorrect" choices), but it is difficult to justify when dealing with latent variables and when there is evidence that people can systematically perform below chance [2]; for example a student who learns the test material incorrectly can systematically perform below chance. Importantly, IRT does not derive its "guessing parameter" from a model of the *m*-AFC task, and SDT suggests there is no need for such a guessing parameter to account for chance performance.

Another difference in how the two approaches model chance performance can be seen with our comparison of SDT to the dichotomous Rasch model. The dichotomous Rasch model provides a good approximation to SDT as long as *m* = 2 and is fixed for all items, and to a lesser degree when *m*>2 and is fixed for all items, but not when *m* varies for different items. This is because chance performance for an IRT model shifts to a different point on the axis when *m* is changed (e.g., if an extra "incorrect" choice is added to an item) while chance performance always lies at *d*′ = 0 in SDT. Intuitively, SDT makes more sense because the difficulty of all *m*-AFC items where a given subject can do no better or worse than pure guessing *should* be the same for that subject.

Mathematically, Eq 3 shows that the dichotomous Rasch model is a special case of SDT when Φ(*x*) is the logistic function, *m* = 2 and *φ*(*x*) is the Dirac delta function, which is represented as *δ*(*x*) and defined as a function that has mass 1 and equals zero at all points except at *x* = 0 where it tends towards +∞. The *sifting property* for *δ*(*x*) says that if any function *f*(*x*) is continuous at *x* = *c*, then $\int_{-\infty }^{+\infty }f(x)\delta (x-c)=f(c)$. Applying the sifting property to Eq 3 with *φ*(*x*) = *δ*(*x*) and Φ(*x*) = *f*(*x*) gives us the dichotomous Rasch model with a "criterion" or "threshold" at *c* = *θ*_*i*_−*b*_*h*_.

This mathematical link between SDT and the dichotomous Rasch model shows that the 2-AFC task is fundamentally different from the task of rating an item 1 or 0. If the task is 2-AFC, then *φ*(*x*) = *δ*(*x*) suggests that the internal response to the correct choice has zero variance which is implausible. If however the task is not 2-AFC and the subject rates the item 1 or 0, then the Dirac delta function has the plausible interpretation of a "criterion" or "threshold" on a rating scale. In general, IRT is inconsistent with the existence of at least two distributions (one for "correct" and at least one for "incorrect") that result from comparing a person's response to an underlying truth state. For this reason, IRT models with their ad hoc adjustments to simulate chance performance should at least in principle not be used to estimate measures from *m*-AFC responses.

We note that our criticism of IRT is restricted to cases where the goal is to *measure* person ability or item difficulty, and this is indeed the case for many applications of IRT in both medical research and educational testing. If however the goal is to *model* the items on a test or how the persons interact with the items, then item discrimination parameters and guessing parameters can have meaning. Nevertheless, the failure of IRT to actually model the *m*-AFC task suggests that it needs further modification before it should be considered preferable to SDT.

Previous studies have extended SDT to latent variables [7,8]. However, these "latent class SDT models" apply to situations where the underlying truth state is unknown and raters are used to estimate the underlying truth state (i.e., the underlying truth state is a latent variable), and the same is true of previous attempts to merge SDT and IRT [9]. Our extension of SDT to latent variables applies to the traditional *m*-AFC task where the experimenter defines the truth state and subject responses are scored with no uncertainty as either "correct" or "incorrect"; however, the item difficulties are unknown and must be estimated from the data. Previous studies have also used SDT to estimate person measures in *d*′ units from forced choice experiments where stimuli were defined by a latent variable, and some of these studies tested the ability of visually impaired individuals to identify emotional expressions or categorize people from images of a person's face [21–23]. The general approach used in these studies was to map a subject's hit and false alarm rates for a set of items to a person measure in *d*′ units for each subject. The problem with this approach is that the estimated person measures are specific to the set of items used, and future studies must use the same set of items if estimated person measures are to be compared to each other. Our innovation is that we estimate both item and person measures on the same scale, which not only allows for direct comparison between persons and items, but also allows researchers to use any subset of items to estimate comparable person measures. For example, our method can be used to create an "item bank" with calibrated item measures for a targeted population from which subsets of items can be chosen to measure changes in patient ability or student ability.
